# Individual Differences in Conditioned Fear and Extinction in Female Rats

**DOI:** 10.3389/fnbeh.2021.740313

**Published:** 2021-08-18

**Authors:** Sarah C. Tryon, Iris M. Sakamoto, Devin M. Kellis, Kris F. Kaigler, Marlene A. Wilson

**Affiliations:** ^1^Department of Pharmacology, Physiology and Neuroscience, University of South Carolina School of Medicine, Columbia, SC, United States; ^2^Columbia VA Health Care System, Columbia, SC, United States

**Keywords:** individual differences, females, freezing, ultrasonic vocalization, fear extinction

## Abstract

The inability to extinguish a traumatic memory is a key aspect of post-traumatic stress disorder (PTSD). While PTSD affects 10–20% of individuals who experience a trauma, women are particularly susceptible to developing the disorder. Despite this notable female vulnerability, few studies have investigated this particular resistance to fear extinction observed in females. Similar to humans, rodent models of Pavlovian fear learning and extinction show a wide range of individual differences in fear learning and extinction, although female rodents are considerably understudied. Therefore, the present study examined individual differences in fear responses, including freezing behavior and ultrasonic vocalizations (USVs), of female Long–Evans rats during acquisition of fear conditioning and cued fear extinction. Similar to prior studies in males, female rats displayed individual variation in freezing during cued fear extinction and were divided into extinction competent (EC) and extinction resistant (ER) phenotypes. Differences in freezing between ER and EC females were accompanied by shifts in rearing during extinction, but no darting was seen in any trial. Freezing behavior during fear learning did not differ between the EC and ER females. Vocalizations emitted in the 22 and 50 kHz ranges during fear learning and extinction were also examined. Unlike vocalizations seen in previous studies in males, very few 22 kHz distress vocalizations were emitted by female rats during fear acquisition and extinction, with no difference between ER and EC groups. Interestingly, all female rats produced significant levels of 50 kHz USVs, and EC females emitted significantly more 50 kHz USVs than ER rats. This difference in 50 kHz USVs was most apparent during initial exposure to the testing environment. These results suggest that like males, female rodents show individual differences in both freezing and USVs during fear extinction, although females appear to vocalize more in the 50 kHz range, especially during initial periods of exposure to the testing environment, and emit very few of the 22 kHz distress calls that are typically observed in males during fear learning or extinction paradigms. Overall, these findings show that female rodents display fear behavior repertoires divergent from males.

## Introduction

The majority of people worldwide experience a traumatic event in their lifetime, yet it is estimated that less than 20% of these individuals develop post-traumatic stress disorder (PTSD) following a life-threatening traumatic event (Kessler et al., 1995; Benish et al., 2008; Ross et al., 2017). A defining feature of PTSD is the inability to extinguish the fear memory, and exposure therapy for clinical treatment of PTSD is based on improving extinction of the fear memory. Unfortunately, the effectiveness of fear extinction through exposure therapy varies drastically among patients (Benish et al., 2008; Holmes and Singewald, 2013; Powers et al., 2015; Fucich et al., 2016). Women are particularly prone to developing PTSD, with a prevalence rate for the disorder twice as high as that of men, even when controlling for type of trauma (Tolin and Foa, 2006; Lebron-Milad and Milad, 2012; Ramikie and Ressler, 2018). This gender disparity suggests a distinctly female vulnerability that may be driven by underlying sex-specific neural substrates, stress responses, and etiologies (Ramikie and Ressler, 2018). Mounting evidence indicates the presence of sexually divergent symptoms, differing anatomical and functional neural processes, and varying stress responses between men and women with PTSD (Felmingham et al., 2010; Inslicht et al., 2013; King et al., 2013; Shvil et al., 2014; Hourani et al., 2015; Birkeland et al., 2017; Cao et al., 2019; Guina et al., 2019; Gay et al., 2020). Understanding how males and females differ behaviorally and neurobiologically in fear learning and extinction is critical for developing more efficacious PTSD treatments for both men and women.

Our lab and others have used preclinical Pavlovian fear learning and extinction models to show that like humans, rodents demonstrate varied levels of fear extinction (Milad and Quirk, 2002; Bush et al., 2007; Galatzer-Levy et al., 2013; Holmes and Singewald, 2013; Shumake et al., 2014; Gruene et al., 2015b; Sharko et al., 2017; Monfils et al., 2019; Kellis et al., 2020). In these models, rodents are exposed to an unconditioned aversive stimulus (US), such as a foot shock, in conjunction with a neutral conditional stimulus (CS), such as a tone. As an associative memory between the CS and US forms, rodents exhibit fearful behaviors such as freezing and 22 kHz distress ultrasonic vocalizations (USVs) in response to the tone. Although the CS initially elicits a fear response on its own, repeated exposure to the conditioned tone in a safe environment without the foot shock promotes extinction of fear responses. The rate and strength of this extinction varies greatly among individuals, comparable to individual differences in treatment responses seen in humans (Milad and Quirk, 2002; Burgos-Robles et al., 2007; Bush et al., 2007; Peters et al., 2010; Galatzer-Levy et al., 2013; Shumake et al., 2014; Gruene et al., 2015a; Sharko et al., 2017; Monfils et al., 2019; Kellis et al., 2020). However, the vast majority of studies examining individual differences in fear conditioning and extinction have utilized males. Within studies using females, however, individual differences have been observed in rates of freezing, darting, and USVs during fear learning or extinction (Shumake et al., 2014; Gruene et al., 2015a; Schwarting, 2018a; Colom-Lapetina et al., 2019).

The understanding of individual differences in fear responses in females is also confounded by sex differences in phenotypic expression of fear. While Pavlovian fear conditioning and extinction in rodents are traditionally measured in terms of freezing, emerging studies show that females display a different repertoire of fear responses compared to their male counterparts (LeDoux, 1994; Shumake et al., 2014; Gruene et al., 2015a,b; Borkar et al., 2020). Female rodents usually exhibit less freezing than males during cued fear learning, conventionally understood as impaired learning, but this interpretation may be confounded by more recent data showing females exhibit darting responses to fear more often than males (Baran et al., 2009; Gruene et al., 2015a; Colom-Lapetina et al., 2019). The existing literature also shows inconsistent sex differences in freezing during fear extinction, with some studies reporting enhanced fear extinction in females compared with males and other studies finding the opposite (Maren et al., 1994; Baran et al., 2009; Milad et al., 2009; Fenton et al., 2014; Velasco et al., 2019; Day and Stevenson, 2020). USVs are another quantitative measure of rodent affective states (Knutson et al., 2002; Sangiamo et al., 2020; Brudzynski, 2021), and are increasingly being used to model affective disorders in rodents (Burgdorf et al., 2020). Long (>300 ms) vocalizations around 22 kHz (ranging from 18 to 32 kHz) are generally emitted during distressing situations including fear conditioning (Borta et al., 2006; Litvin et al., 2007), whereas shorter (20–80 ms) vocalizations around 50 kHz (32–70 kHz) are produced in a variety of rewarding conditions such as positive social interactions and administration of addictive drugs (Portfors, 2007; Schwarting and Wöhr, 2012; Brudzynski, 2015, 2021). Given the correlation between emission of 50 kHz USVs and approach behavior, these vocalizations are deemed reflective of positive affect, and are not generally reported during exposure to fearful stimuli. Sex differences have been reported in the emission of these USVs (Kosten et al., 2006; Shumake et al., 2014; Kisko et al., 2021; Willadsen et al., 2021).

Thus, complete understanding of affective states and their underlying mechanisms must take all fear behaviors displayed by each sex into consideration (Brudzynski, 2001), because it is likely that distinct neural circuits underlie these different fear responses (Koo et al., 2004; Shumake et al., 2014). Further, less than 2% of fear conditioning and extinction studies have examined females, indicating that this body of literature is substantially lacking in sex-specific and female data (Lebron-Milad and Milad, 2012). Preclinical rodent studies have not consistently reported distinct extinction phenotypes in females (Shalev et al., 2017; Ramikie and Ressler, 2018), perhaps because models that exclusively quantify freezing are not sensitive to female-specific phenotypes of fearful behavior. To address this gap, the present study examined freezing and other behaviors, as well as both 22 and 50 kHz USVs, in a sample of female rats during a fear conditioning and cued fear extinction paradigm. Based on the reviewed literature and our previous findings in Long–Evans male rats (Sharko et al., 2017; Kellis et al., 2020), we hypothesized that like males, female rats would exhibit individual differences in fear extinction responses, presenting either extinction competent (EC) or extinction resistant (ER) phenotypes. We predicted these individual differences in fear might be observed not only in freezing, but also in more affective differences in 22 and 50 kHz USVs and/or other behaviors such as rearing, grooming, or darting. Our findings demonstrate that female Long–Evans rats, like males, display individual differences in freezing and rearing behaviors during cued extinction learning and recall. We also find that whereas females emit a low number of 22 kHz distress calls, they emit a high number of 50 kHz USVs, particularly during initial exposure to the testing environment during fear learning and extinction trials. Interestingly, EC females show more of these 50 kHz USVs than ER females, and 50 kHz USV emissions prior to fear learning predicts freezing during extinction learning.

## Materials and Methods

### Subjects

Adult female outbred Long–Evans rats (∼60 days of age, 150–175 g on arrival; Envigo, Indianapolis, IN, United States) were individually housed in a climate-controlled vivarium and maintained on a 12-h light/dark cycle with *ad libitum* access to food and water. All animals were treated in compliance with the American Association for Laboratory Animal Science (AALAS) and all procedures were approved by the University of South Carolina’s Institutional Care and Use Committee. Animals were handled daily for 2 weeks prior to behavioral testing. To assess estrous cycle phase, vaginal lavages were performed daily for 2 weeks before behavioral testing started and continued until the conclusion of testing. Vaginal lavage was performed after behavioral testing each day to avoid additional handling stress (Wilson et al., 2004; Finnell et al., 2018). Vaginal cytology was determined daily by examining fresh cytology samples under a microscope. Slides were then fixed with 95% ethanol and stained with hematoxylin and eosin for subsequent verification by an additional observer [see Finnell et al. (2018) supplemental information for detailed methods]. Uterine weights were measured following euthanasia to confirm the day of the estrous cycle on the final day of testing.

### Fear Conditioning and Extinction Procedures

**FIGURE 1:**
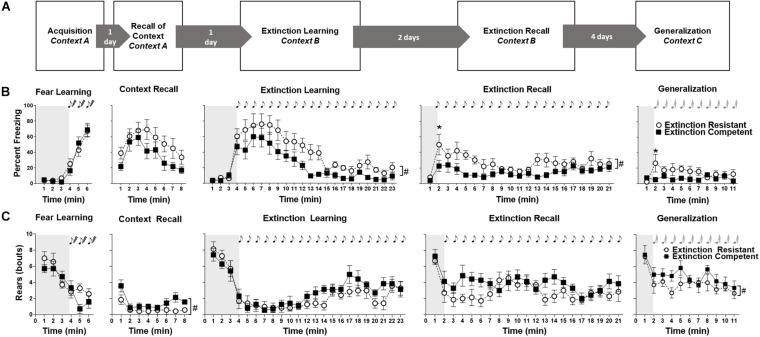
Testing protocol is shown in panel **(A)**. Freezing **(B)** and rearing **(C)** behaviors in extinction competent (EC) and extinction resistant (ER) rats are shown during fear acquisition, contextual recall, extinction learning, extinction recall, and generalization trials. Shaded area represents the unconditioned period before the tone–shock pairings (fear learning) or the first conditioned/novel tone (extinction learning, extinction recall, and generalization). Freezing behavior is expressed as percent freezing in 1-min bins while rearing is the number of rears during each minute. EC and ER rats showed no differences in freezing **(A)** during acquisition or context recall, but ER rats displayed higher freezing in response to the CS+ (tone) than EC rats during extinction learning and extinction recall. A time by phenotype interaction in freezing was seen during generalization since ER rats exhibited higher freezing than EC rats, but only during the first novel tone presentation during this trial. Bouts of rearing **(C)** decreased across time during all trials as demonstrated by main effects of time, since rearing was high during periods before the tone presentation (shaded areas) and decreased as freezing behavior increased. Overall differences in rearing bouts between ER and EC groups were only seen during contextual fear extinction, and generalization. Points represent mean ± SEM, with *N* = 7 ER and *N* = 7 EC rats per group. ^#^*P* < 0.05 for main ER–EC difference; *P < 0.05 significant differences at each time point. Tones are denoted by the notes.

Experimental procedures were adapted from our previous protocols in males demonstrating individual differences in fear extinction (Sharko et al., 2017; Kellis et al., 2020; Figure 1A). For fear acquisition on day 1, rats were placed in a shock box (Context A) within a sound-attenuating chamber containing a ventilation fan, speaker, and a house light (CleverSys, Inc., Reston, VA, United States), with a microphone for recording USVs below the box (Noldus Information Technology, Leesburg, VA, United States). Mild (7%) ammonium hydroxide was used to clean the shock box between animals. Unconditioned freezing, behavioral responses, and USVs were recorded for the first 3 min. Beginning at the third minute, animals were presented with three tone–shock pairings consisting of a 10 s tone (2 kHz, 80 dB) that co-terminated with a shock (1 mA, 1 s) with a 60 s inter-tone-interval. On day 2, context recall was assessed by placing rats back in Context A for 8 min in the absence of tones or shocks, during which freezing, additional behavioral responses, and USVs were recorded. On day 3, animals were placed in a novel environment (Context B) for cued extinction learning. Context B consisted of a round bottom Plexiglas bowl with aspen bedding and lemon scent, with the microphone for USV recording fixed to the upper side of the bowl. Context B was cleaned with ethanol (70%) between subjects and was located in a sound attenuated chamber distinct from Context A. Unconditioned responses were recorded for the first 3 min in Context B, after which animals were presented with 20 conditioned tones (2 kHz, 10 s, 80 dB) without shock at 1 min intervals, for a total testing period of 23 min. After 48 h (day 5 of testing), the rats were returned to Context B to assess extinction recall. After 1 min, they were presented with another 20 tone presentations (2 kHz, 10 s, 80 dB). On day 8 of testing, to assess generalization of fear learning, rats were placed in a cylindrical glass bowl in a novel sound-attenuated chamber with a distinct floor of paper pads and vanilla scent (Context C). A 1 min period to assess unconditioned freezing and USVs was followed by 10 novel unconditioned tones (3.5 kHz, 10 s, 80 dB) at 1 min intervals. After behavioral testing on day 8, vaginal lavage for estrous cycle assessment was performed and rats were then euthanized to determine uterine weights.

### Behavioral Analysis

Freezing behavior during all trials was evaluated in 1-min bins using FreezeScan software (CleverSys, Inc., Reston, VA, United States). Automatic software parameters were set to detect freezing as the absence of movement excluding respiration. Percent freezing was calculated as the percent of time spent freezing in each 1-min bin that included each tone and the following inter-tone interval. As described previously for males (Sharko et al., 2017; Kellis et al., 2020), females were divided into ER (high freezing) and EC (low freezing) groups using a median split of average freezing during the last 10 min (10 tones) of extinction learning.

Overt behaviors were also manually scored for all trials from video recordings using Noldus^®^ Observer XT software (Noldus Information Technology, Leesburg, VA, United States) by an observer who was blinded to the phenotype of the animal. The following behaviors were quantified by hand scoring: the number of grooming, rearing, and burying events, and the duration of burying, grooming and freezing; burying was only seen and scored in Contexts B and C, since the shock box floor was a metal grid. Grooming was classified as forelimb movements to the face, trunk, and genital regions. Rearing was classified as movements in which the animal was upright with only the two hind legs touching the floor. Freezing was classified as the cessation of all movements except breathing. Burying, which was seen in Contexts B and C, was defined as moving the bedding on the cage floor with the paws or head. No darting was observed during any trial [as described in Gruene et al. (2015a)]. It was also noticed that some animals investigated the microphone used for recording USVs at the top of Contexts B and C while they were rearing, so these events were also scored. Freezing from manual (Observer^®^ XT) and automated (FreezeScan) methods were similar, so all data presented are from the automated freezing data using FreezeScan.

Ultrasonic vocalizations were recorded using an ultrasonic microphone (full spectrum, USB port, 250,000 samples per second and 16 bit resolution) with UltraVox XT software version 3.2.106/3.2.108 (Noldus Information Technology, Leesburg, VA, United States). During fear acquisition and context recall trials (Context A), microphones were placed below the shock chamber floor. During extinction learning, extinction recall, and generalization trials in Contexts B and C, microphones were oriented above the rats and fastened to one side of the experimental chamber. USVs in the 22 and 50 kHz range were manually labeled on a spectrogram (SFT length = 2048, Zero Padding = 1, Overlap = 90%) in the Analysis/Call Labeling tab, which provided quantitative information including call duration (ms), call start and stop time, peak frequency (frequency at maximum amplitude, Hz), and mean amplitude for each call. Data was then exported into Microsoft Excel for analysis of USV parameters in 1 min bins to correlate with behavioral analysis.

### Statistical Analyses

Extinction resistant and EC groups were determined using a median split of freezing during the last 10 tones of extinction learning (as described previously for males) (Sharko et al., 2017; Kellis et al., 2020). Freezing behavior during each trial was recorded and calculated by FreezeScan software as percent freezing per 1 min time bin. Percent time spent freezing, grooming, or burying, plus rearing, grooming, and burying events, during each 1 min bin over each trial were compared using a one-way analysis of variance (ANOVA; ER versus EC) with repeated measures (time), although only rearing data is shown over time. The total number or percent time spent in each behavior during unconditioned and conditioned periods of fear learning and extinction trials were also analyzed using a three-way ANOVA to compare extinction phenotypes, behaviors, and conditioned versus unconditioned periods. Specific differences between groups were assessed using Bonferroni *post hoc* analysis.

The number and total duration (data not reported) of 22 and 50 kHz USVs between ER and EC groups were also analyzed over time using a one-way ANOVA with repeated measures (time), and 22 and 50 kHz USVs were analyzed separately for each trial. For the 22 and 50 kHz USV data, the total number of calls, average duration per call, average peak frequency of calls, and mean amplitude of calls in each trial were also analyzed using two-way ANOVA to compare overall ER versus EC differences between all behavioral trials. Because significant 50 kHz USVs were seen during exposure to the novel contexts, the total number of calls during the 3 min period of unconditioned vocalizations versus conditioned USVs in the acquisition and extinction learning trials were also compared between ER and EC groups using two-way ANOVA. For all analyses, specific differences between groups were assessed using Bonferroni *post hoc* analysis, and due to the variability in calls between groups (with many animals not showing USVs) the Geisser–Greenhouse correction was used for non-normally distributed data. All statistical analysis was performed using GraphPad Prism 8.0 (GraphPad Software, San Diego, CA, United States). Significance level for all analyses was set at *P* < 0.05.

## Results

### Freezing Behavior in Females

To allow comparisons with our previous results in male Long–Evans rats (Sharko et al., 2017; Kellis et al., 2020), female rats were classified as ER and EC phenotypes based on percent freezing during the last 10 min (10 tones) of cued fear extinction. Using a median split, high-freezing animals were classified as ER (*N* = 7) and low-freezing animals were classified as EC (*N* = 7). Figure 1B shows freezing over time in the ER and EC groups during fear learning, context recall, extinction learning, extinction recall, and generalization to a novel tone. ER and EC rats showed no differences in freezing behavior during fear learning [Figure 1B; *F*(1,12) = 0.03, *P* = 0.9]. There was a significant effect of time [*F*(5,60) = 58.23, *P* < 0.0001], but no time × group interaction [*F*(5,60) = 0.73, *P* = 0.61]. The effect of time was related to the low level of unconditioned freezing during the first 3 min, and the gradual increase in freezing with successive tone–shock pairings during acquisition of fear learning. Similarly, during contextual fear recall trials, there was no difference in freezing between ER and EC groups [*F*(1,12) = 2.9, *P* = 0.12]. There was a significant effect of time [*F*(7,84) = 8.65, *P* < 0.0001], but no significant time × group interaction [*F*(7,84) = 0.57, *P* = 0.78].

Unlike fear learning and contextual fear recall, ER rats froze significantly more than EC rats during extinction learning [*F*(1,12) = 8.13, *P* < 0.015]. Both groups displayed significant differences in freezing over time [*F*(22,264) = 18.12, *P* < 0.0001], although the time × ER/EC interaction was not significant [*F*(22,264) = 0.87, *P* = 0.63]. This demonstrates that females show distinct extinction phenotypes similar to what has previously been observed by our lab and others in males (Bush et al., 2007; Burgos-Robles et al., 2009; Galatzer-Levy et al., 2013; Holmes and Singewald, 2013; Shumake et al., 2014; Gruene et al., 2015a; Sharko et al., 2017; Kellis et al., 2020), although the difference between ER and EC phenotypes in freezing behavior during extinction learning in females observed here was less pronounced than what we have observed in males (Sharko et al., 2017; Kellis et al., 2020). For extinction recall, ER rats froze significantly more than EC rats [*F*(1,12) = 15.52, *P* = 0.002], and both phenotypes showed a significant difference in freezing over time [*F*(20,240) = 2.72, *P* = 0.0002], but the time × group interaction was not significant [*F*(20,240) = 0.99, *P* = 0.47]. *Post hoc* Bonferroni analysis indicated the ER group froze significantly more than the EC group after presentation of the first cue during extinction recall, but this divergence rapidly disappeared during subsequent cue presentations.

During generalization trials where rats were exposed to a novel tone in a new Context C (Figures 1A,B), there was no significant difference in freezing between ER and EC rats [*F*(1,12) = 3.09, *P* = 0.10] and no main effect of time [*F*(10,120) = 1.46, *P* = 0.16]. There was, however, a significant group × time interaction, with ER rats freezing significantly more than EC females during the first novel tone presentation (minute 2) of the trial [*F*(10,120) = 2.53, *P* < 0.009], which might suggest a slight difference in generalization between ER and EC groups.

Although we tracked the stage of the estrous cycle in the study, the females were at random stages of the cycle during all the behavioral testing. All animals showed normal 4 (*N* = 11) or 5 (*N* = 3) day estrous cycles. Both the ER and EC groups included animals on different days of the cycle during each stage of the fear learning and extinction paradigm, suggesting that the differences in freezing and USVs between ER and EC groups could not be solely based on hormone fluctuations during the cycle. For example, on the day of extinction learning the ER group had two rats in metestrous, three rats in diestrous, one rat in proestrous, and one rat in estrous, while the EC group had four rats in metestrous, two rats in diestrous, and one rat in estrous. Further, behavioral testing was conducted in the early light period of the light:dark cycle, which would have minimized some of the gonadal hormone fluctuations.

### Other Behaviors in Females

**FIGURE 2:**
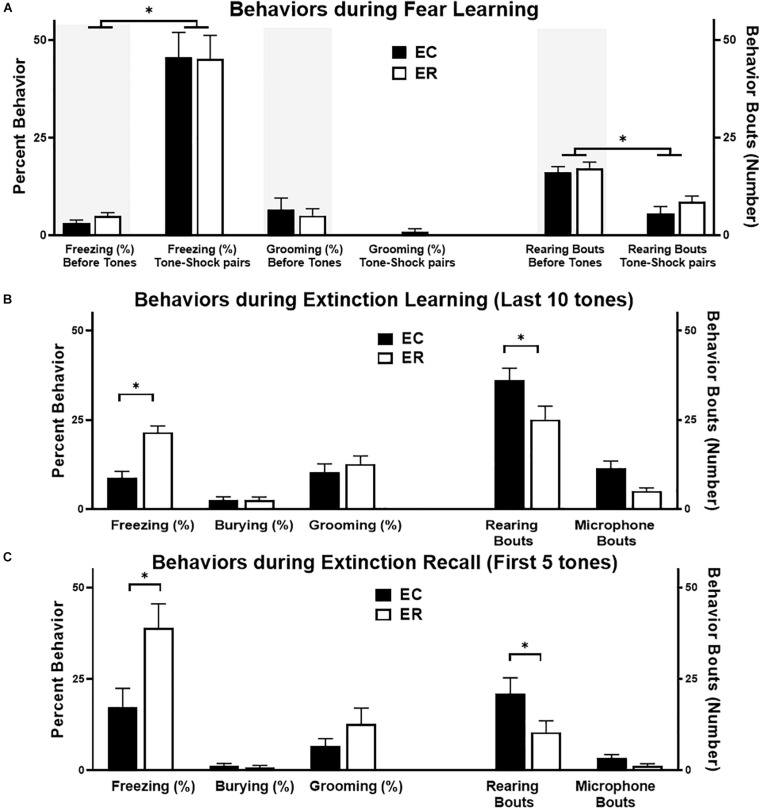
Summary of behaviors in extinction competent (EC; black bars) and extinction resistant (ER, open bars) female rats during fear learning **(A)**, the last 10 tones during extinction learning **(B)**, and the first 5 tones of extinction recall **(C)**. The left *Y* axis is for the percent of time spent freezing, grooming, or burying, while the right *Y* axis is for the number of rearing bouts or microphone interactions. Percent freezing is from automated analysis with FreezeScan, while other behaviors were hand-scored using Observer^®^ XT. During fear learning **(A)**, both the 3 min prior to conditioning (shaded areas) and the 3 min during tone–shock pairings are shown. Both ER and EC rats display similar increases in freezing alongside a decrease in rearing during the tone–shock pairings phase of fear learning. During both extinction learning **(B)** and extinction recall **(C)** ER females froze significantly more than EC rats, and ER rats displayed fewer rears than EC females during this same period. Bars represent mean ± SEM, with *N* = 7 ER and *N* = 7 EC rats per group. ^∗^*P* < 0.05.

Other behaviors were manually scored and quantified for all trials using Observer^®^ XT. These included rearing, grooming, and burying, plus investigation of the microphone used for USV recording, which was similar to rearing. Of note, no darting behavior was observed during any trial, despite observations of such behavior previously observed by Gruene et al. (2015a). The amounts of grooming and burying behaviors were low and the changes over time are not shown, although the summarized data for fear learning, extinction learning, and extinction recall are seen in Figure 2. Rearing events in EC and ER groups for each trial are shown in Figure 1C. Similar to what has been reported in males (Wöhr et al., 2005), we found that rearing behavior was high during the period before tone–shock pairings in fear learning and the period prior to presentation of the conditioned tones in extinction trials (see shaded areas of Figure 1), and rearing decreased as freezing behavior increased (Figure 1). No differences in rearing were seen between EC and ER groups during acquisition [*F*(1,12) = 1.45, *P* = 0.25], extinction learning [*F*(1,12) = 1.38, *P* = 0.26], or extinction recall [*F*(1,12) = 3.27, *P* = 0.10] (Figure 1C). For these three trials, there was a significant effect of time on rearing [*F*(5,60) = 16.68 for *P* < 0.0001, fear learning; *F*(22,264) = 15.71 for *P* < 0.0001, extinction learning; *F*(20,240) = 3.56, *P* < 0.0001 for extinction recall] that is related to the high number of rears before presentation of the tones followed by a decrease in rearing after tone presentations. However, there was no time × phenotype interaction for any of these three trials [*F*(5,60) = 1.80, *P* = 0.13, for fear learning; *F*(22,264) = 0.95, *P* = 0.53 for extinction learning; *F*(20,240) = 1.23, *P* = 0.23 for extinction recall]. During contextual recall, however, EC rats exhibited significantly more rears during the trial than ER rats [*F*(1,12) = 6.57, *P* < 0.05], and both EC and ER groups showed a decrease in rearing over time [*F*(7,84) = 5.27, *P* < 0.0001], although there was no time × phenotype interaction [*F*(7,84) = 0.94, *P* = 0.5]. Similarly, during generalization to a novel tone, EC rats reared significantly more than ER rats [*F*(1,11) = 6.36, *P* < 0.05]. Both groups displayed a reduction in rearing over the generalization trial [*F*(10,110) = 4.76, *P* < 0.0001], although the time × phenotype interaction was not significant [*F*(10,110) = 0.56, *P* = 0.85].

As summarized in Figure 2A, during fear learning the percent of time freezing increased but grooming time did not change during the tone–shock pairings, particularly when compared with unconditioned freezing during the initial 3 min period in the new context [*F*(1,48) = 58, *P* < 0.0001]. The number of rearing bouts decreased during tone–shock pairings, however, ER and EC groups did not differ in any of these behaviors (freezing, grooming, or rearing) during the fear learning trial. In comparison, during extinction learning and extinction recall trials (Figure 2 middle and bottom panels), ER and EC groups differed in behavioral patterns, with the ER group showing greater percent time freezing than the EC group during the last 10 tones of extinction learning [*F*(1,36) = 12.3, *P* = 0.001] and the first 5 tones of extinction recall [*F*(1,36) = 7.9, *P* = 0.008], but a lower number of rearing bouts during these same trials [*F*(1,24) = 9.7, *P* < 0.005 for ER–EC effect during extinction learning and *F*(1,24) = 5.5, *P* < 0.03 for extinction recall]. This suggests that the primary behavior differing between the phenotypes when they are not freezing is rearing, which is supported by the significant negative correlation between freezing and rears during extinction learning (see Figure 5C; *r* = −0.76, *R*^2^ = 0.6, *P* < 0.002).

**FIGURE 3:**
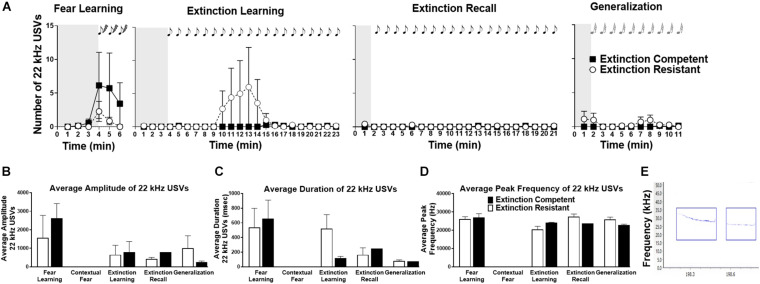
Female rats emitted very few 22 kHz ultrasonic vocalizations (USVs) during fear learning, extinction learning, extinction recall, and generalization trials, and no 22 kHz USVs were seen during contextual fear recall (not shown). In panel **(A)**, the number of 22 kHz USVs in extinction competent (filled squares, EC) and extinction resistant (open circles, ER) rats in each 1-min time bin is shown. Shaded areas represent the unconditioned vocalizations before tone–shock pairings (fear learning) or the first conditioned/novel tone (extinction learning, extinction recall, and generalization). Very few females showed 22 kHz vocalizations, and most of these were during fear learning (tone–shock pairings). EC and ER rats did not differ significantly in the number of 22 kHz calls during any of the trials. Panels **(B–D)** show that EC and ER phenotypes did not show any differences in the acoustic parameters of 22 kHz USVs, including the average amplitude of each call **(B)**, average duration of each call **(C)**, or average peak frequency of the calls **(D)** across all trials. Panel **(E)** shows a representative spectrogram of 22 kHz USVs; the blue boxes identify the detected 22 kHz USVs using UltraVox software. Points and bars represent mean ± SEM, with *N* = 7 ER and *N* = 7 EC rats per group.

**FIGURE 4:**
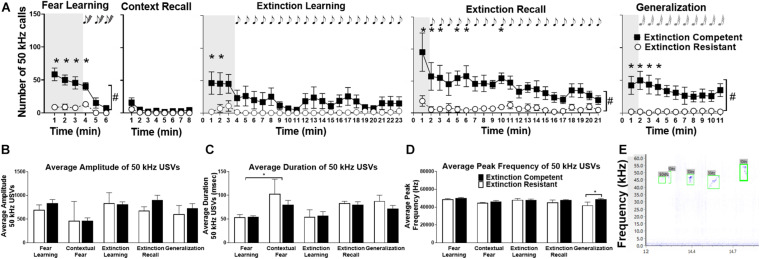
Female rats emitted significant numbers of 50 kHz ultrasonic vocalizations (USVs) during fear learning, extinction learning, extinction recall, and generalization trials, although fewer 50 kHz USVs were seen during contextual fear recall. Panel **(A)** shows extinction competent (filled squares, EC) females emitted a significantly greater number of 50 kHz USVs compared to extinction resistant (open circles, ER) rats over time. The shaded areas represent the unconditioned vocalizations before tone–shock pairings (fear learning) or the first conditioned/novel tone (extinction learning, extinction recall, and generalization). During acquisition, EC rats emit significantly more 50 kHz USVs than the ER rats predominantly during the first 3 min of exposure to Context A prior to tone–shock pairings; 50 kHz USVs decreased during fear acquisition and the tone–shock pairings. Similar to acquisition, EC rats emit more 50 kHz USVs than ER rats during extinction learning, extinction recall, and generalization trials, and the number of 50 kHz USVs was greatest during periods before the tone presentations and decreased over time in these trials. Panels **(B–D)** show that acoustic parameters of 50 kHz USVs were similar in EC and ER phenotypes including the average amplitude of each call **(B)**, average duration of each call **(C)**, or average peak frequency of the calls **(D)** across all trials, with the exception of an ER–EC difference in frequency during generalization. Panel **(E)** shows a representative spectrogram of 50 kHz USVs; the green boxes identify the detected USVs using UltraVox software. Points and bars represent mean ± SEM, with *N* = 7 ER and *N* = 7 EC rats per group. ^#^*P* < 0.05 main effect of phenotype; *P < 0.05 significant phenotypic differences at 1-min bins).

**FIGURE 5:**
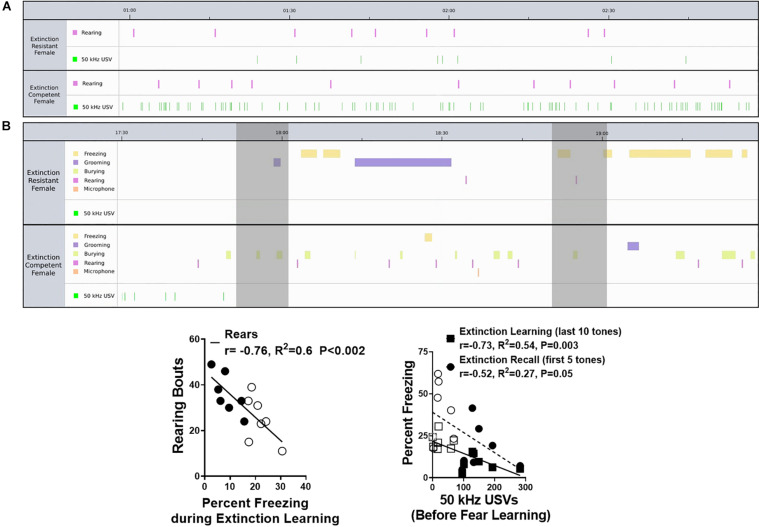
Representative ethograms from extinction resistant (ER) and extinction competent (EC) females comparing behaviors and ultrasonic vocalizations (USVs) prior to fear learning **(A)** and during extinction learning **(B)**. Ethograms in panel **(A)** show 2 min prior to the tone–shock pairings demonstrating similar numbers of rearing bouts (pink) in the ER and EC rats, but significantly more 50 kHz USVs emitted by the EC female compared to the ER rat during the initial exposure to Context A. Ethograms in panel **(B)** show freezing (yellow), grooming (purple), burying (light green), rearing (pink), microphone exploration (orange), and 50 kHz USVs (green) in an ER and EC female during 2 min of tone presentation (shaded bars) during extinction learning; this represents tones number 15 and 16 during the last 5 min of extinction learning. Note that the ER female shows more freezing, but less rearing, burying, and 50 kHz USVs, compared to the EC female during this same period of the trial. Panel **(C)** demonstrates the number of rearing bouts is negatively correlated with the percent freezing during extinction learning. Panel **(D)** shows the number of 50 kHz USVs during the 3 min prior to fear learning is negatively correlated with the percent freezing during extinction learning and extinction recall, suggesting more 50 kHz USVs during the initial exposure to Context A (prior to any fear conditioning) is associated with better extinction (less freezing) during extinction in females. *N* = 7 ER and *N* = 7 EC rats per group. Closed symbols are EC females and open symbols are ER females.

### 22-kHz Ultrasonic Vocalizations in Females

Ultrasonic vocalizations in the 22 kHz range emitted by ER and EC female rats were also assessed. As seen in Figure 3A, females emitted a low level of 22 kHz USVs (“distress calls”) during fear learning, extinction learning, extinction recall, and tone generalization, and no calls were seen during re-exposure to the context (data not shown). During fear learning, only 43% of ER rats vocalized in the 22 kHz range, compared to 57% of the EC rats. However, despite the different number of EC and ER rats that vocalized in this range, during fear learning there was no significant difference in the total number of 22 kHz calls between ER and EC rats [*F*(1,12) = 0.90, *P* = 0.36], no significant effect of time [*F*(5,60) = 1.90, *P* = 0.11], and no time × ER/EC interaction [*F*(5,60) = 0.69, *P* = 0.63] (Figure 3A). During extinction learning only a subset of ER and EC rats (43% of rats in each group) vocalized (Figure 3A). A two-way ANOVA did not show any significant effects of extinction phenotype [*F*(1,12) = 0.97, *P* = 0.34], time [*F*(22,264) = 0.96, *P* = 0.52], or time × phenotype interaction [*F*(22,264) = 1.00, *P* = 0.46] on the total number of 22 kHz calls during extinction learning. Emission of 22 kHz USVs was largely absent in both groups during extinction recall, and no significant differences in number of 22 kHz calls between ER and EC rats [*F*(1,12) = 1.23, *P* = 0.29], no effect of time [*F*(20,240) = 1.16, *P* = 0.29], and no time × group interaction [*F*(20,240) = 1.39, *P* = 0.13] were observed. Finally, generalization trials also showed no significant effects of group [*F*(1,12) = 1.28, *P* = 0.28], time [*F*(10,120) = 0.87, *P* = 0.56], or time × group interaction [*F*(10,120) = 1.02, *P* = 0.43] in the number of 22 kHz USVs.

We also compared acoustic parameters across all trials for 22 kHz calls (Figures 3B–E shows representative 22 kHz USVs). As a whole, likely due to the overall low number of 22 kHz emissions, most of the results comparing 22 kHz USVs for extinction phenotypes and trials were not significant. Mean call amplitude was not significantly different between trials [*F*(3,15) = 1.85, *P* = 0.18], between ER/EC phenotypes [*F*(1,15) = 0.11, *P* = 0.75] and there was no significant group × trial interaction [*F*(3,15) = 0.45, *P* = 0.72]. There were no significant differences in average call duration across trials [*F*(3,14) = 1.73, *P* = 0.21], between ER/EC phenotypes [*F*(1,14) = 0.07, *P* = 0.79], nor was there a group × trial interaction [*F*(3,14) = 0.60, *P* = 0.63]. Average peak frequency was not significantly different between trials [*F*(3,15) = 2.21, *P* = 0.13], nor was there an ER/EC main effect [*F*(1,15) = 0.12, *P* = 0.73], nor group × trial interaction [*F*(3,15) = 1.54, *P* = 0.24].

### 50-kHz Ultrasonic Vocalizations in Females

Compared to 22 kHz calls, female rats showed more USVs in the 50 kHz range, and there were differences between ER and EC groups in several trials. The overall number of 50 kHz vocalizations was high during fear learning prior to the tone–shock pairings, with 100% of rats producing 50 kHz USVs. Rats emitted most 50 kHz USVs during the first 3 min in Context A during the period used to assess unconditioned freezing (shaded region in Figure 4A), and these calls decreased during the tone–shock pairings. Interestingly, compared to ER rats, EC rats produced significantly more 50 kHz USVs during minutes 1–3 (prior to tone–shock pairings), and during the first conditioned tone (Figure 4A), as well as in total throughout the trial. For fear learning, there was a significant main effect of extinction phenotype [*F*(1,12) = 45.04, *P* < 0.0001], time [*F*(5,60) = 11.41, *P* < 0.0001], and time × ER/EC interaction [*F*(5,60) = 4.83, *P* < 0.001] on the number of 50 kHz calls. During contextual fear recall (when animals were exposed to the conditioned context again without tones or shocks) the total number of 50 kHz calls was low relative to other trials. During context recall, there was a significant effect over time [*F*(1.13,13.59) = 5.4, *P* < 0.035] but no effects of extinction phenotype [*F*(1,12) = 1.8, *P* = 0.2] and no ER/EC × time interaction [*F*(7,84) = 0.96, *P* = 0.5] on number of 50 kHz USVs (Figure 4).

During extinction learning, 86% of the females vocalized in the 50 kHz range, with 71% of ER rats and 100% of EC rats vocalizing in this high frequency range. A two-way ANOVA demonstrated a significant effect of time [*F*(22,264) = 3.96, *P* < 0.0001] and a time × ER/EC interaction [*F*(22,264) = 2.34, *P* < 0.001] on the total number of 50 kHz calls during fear extinction, but there was no significant main effect of ER/EC phenotype [*F*(1,12) = 3.59, *P* = 0.08]. The effect of time was due to a higher number of 50 kHz USVs during the first 3 min of the trial in a new environment (Context B) prior to tone presentation, similar to the pattern seen during fear learning. In extinction recall trials, EC rats also produced more 50 kHz calls than ER rats despite being returned to Context B. There was a significant main effect of extinction phenotype [*F*(1,12) = 17.8, *P* < 0.002], time [*F*(20,240) = 2.81, *P* = 0.0001], and a phenotype × time interaction [*F*(20,240) = 1.62, *P* < 0.05] on total number of calls. Similar to acquisition and extinction learning trials, in generalization trials when rats were in a new context and exposed to novel (unconditioned) tones, EC rats vocalized significantly more than ER rats in the 50 kHz range. Examining total number of calls, there was a significant main effect of extinction phenotype [*F*(1,12) = 10.10, *P* < 0.008], time [*F*(10,120) = 2.00, *P* < 0.04], and phenotype × time interaction [*F*(10,120) = 2.52, *P* < 0.009] during generalization.

Figures 4B–D demonstrate a comparison of different call parameters across trials for 50 kHz calls, and Figure 4E shows representative 50 kHz USVs. For average 50 kHz call amplitude, there were no significant differences between ER/EC groups [*F*(1,51) = 1.39, *P* = 0.25], between trials [*F*(4,51) = 1.88, *P* = 0.13], or group × trial interaction [*F*(4,51) = 0.35, *P* = 0.85]. For average 50 kHz call duration, there was a main effect of trial [*F*(4,51) = 6.70, *P* = 0.0002] due to a significant difference between fear learning and context recall trials. However, there was no significant ER/EC difference [*F*(1,51) = 1.69, *P* = 0.20] nor was there a group × trial interaction [*F*(4,51) = 0.65, *P* = 0.63] for average duration per 50 kHz USV. For average peak frequency, there was a significant ER/EC difference [*F*(1,51) = 4.21, *P* = 0.045], but *post hoc* Bonferroni comparisons showed this difference to be in the generalization trial where both the context and tones were novel. There were no significant effects of trial [*F*(4,51) = 1.96, *P* = 0.11] or group × trial interaction [*F*(4,51) = 1.23, *P* = 0.31] for the average peak frequency of 50 kHz calls.

Overall, these results suggest that EC females emit more 50 kHz USVs during initial exposure to the testing context than their ER counterparts. Given the high number of 50 kHz USVs during the first 3 min of both the fear learning and extinction learning trials, we compared the total number of 50 kHz USVs during this period in both trials using a two-way ANOVA (data not shown). The mean number of 50 kHz USVs for the ER group during fear acquisition (26 ± 10 calls) and extinction learning (20 ± 15 calls) were similar, but significantly less than those of the EC group during acquisition (154 ± 25 calls) and extinction learning (136 ± 46 calls). This resulted in a main effect of extinction phenotype for number of calls [*F*(1,24) = 19.50, *P* = 0.0002], but no difference between the trials [*F*(1,24) = 0.18, *P* = 0.67] and no interaction [*F*(1,24) = 0.05, *P* = 0.83]. As seen in Figure 5D, there was also a significant negative correlation between the number of 50 kHz USVs produced during this initial exposure to Context A before fear learning and the percent freezing during extinction learning (*r* = −0.73, *R*^2^ = 0.54, *P* < 0.003 for extinction learning). The different patterns in freezing, rearing, grooming, and 50 kHz vocalizations between rats representative of the ER and EC groups are illustrated in the ethograms shown in Figure 5. The top ethogram (5A) illustrates the marked difference in 50 kHz USVs between representative ER and EC rats prior to fear learning while exploring the novel context, while the bottom ethogram (5B) shows the higher level of freezing in ER rats compared with EC rats during a 2 min period of the last 10 min of extinction learning.

## Discussion

The current study examined behavioral responses and USVs to learned fear and fear extinction in female Long–Evans rats using a Pavlovian cued fear conditioning and extinction paradigm. Individual differences in extinction of learned fear have been previously shown in male rodents in our lab (Sharko et al., 2017; Kellis et al., 2020) and other labs (Milad and Quirk, 2002; Burgos-Robles et al., 2007, 2009; Bush et al., 2007; Galatzer-Levy et al., 2013; Holmes and Singewald, 2013; Shumake et al., 2014; Gruene et al., 2015b; King et al., 2018; Monfils et al., 2019) with males exhibiting a wide range of high and low extinction. Only a few studies to date have characterized extinction phenotypes in females, with conflicting results (Pryce et al., 1999; Kosten et al., 2005; Baran et al., 2009, 2010; Fenton et al., 2014; Shumake et al., 2014; Gruene et al., 2015a; Clark et al., 2019). Our results contribute novel findings to this growing literature that demonstrate females also display individual differences in freezing, rearing, and USVs (especially in the 50 kHz range) during cued fear extinction and extinction recall.

The current study investigated multiple behavioral indices of fear in a female cohort of outbred Long–Evans rats. In a manner similar to what has been reported in the literature in males (Milad and Quirk, 2002; Burgos-Robles et al., 2007, 2009; Bush et al., 2007; Galatzer-Levy et al., 2013; Holmes and Singewald, 2013; Shumake et al., 2014; Gruene et al., 2015a,b; Sharko et al., 2017; King et al., 2018; Monfils et al., 2019; Kellis et al., 2020), we found female rats to exhibit individual differences in freezing behavior during extinction learning, such that females could be divided into EC and ER phenotypes. Like our previous studies (Sharko et al., 2017; Kellis et al., 2020) and other reports (Milad and Quirk, 2002; Burgos-Robles et al., 2007, 2009; Bush et al., 2007; Galatzer-Levy et al., 2013; Shumake et al., 2014) in males, ER and EC females did not show significant differences in freezing during fear learning, but differences emerged during cued fear extinction learning. Similar to prior studies in males, this suggests phenotypic differences are not due to the initial fear-associated freezing during acquisition or differences in sensitivity to the foot-shocks between groups, but rather that the differences rely more on the neural mechanisms associated with extinction learning and/or recall. Interestingly, however, the divergence between the ER and EC freezing phenotypes was less robust in females in the present study than the ER–EC difference we have previously observed in males (Sharko et al., 2017; Kellis et al., 2020). This was similar to observations by Shumake et al. (2014) that there was a bimodal distribution in freezing during extinction in males but not females.

The present study also investigated other overt behaviors in female rats, as previous studies have shown females exhibit a different repertoire of fear behaviors during fear conditioning and extinction than males (Gruene et al., 2015a,b). Similar to what has been reported in the literature in male rodents, females in our study displayed reduced rearing behavior following tone presentation during fear acquisition and an increase in rearing during extinction learning (Wöhr et al., 2005). Rather expectedly, EC rats froze significantly less than ER rats during the last 10 tones of extinction learning, and EC rats likewise exhibited more rearing during this same time period compared to their ER counterparts, suggesting that rearing accounts for most of the active behavior during the trials when females were not freezing. This was supported by a significant negative correlation between the percent of freezing and rearing bouts during extinction learning. It has previously been reported that females displaying higher darting behavior during acquisition also displayed lower freezing during extinction recall that was not a result of increased darting during this same trial, suggesting that darting behaviors are a separate phenotype that could be predictive of extinction learning competency (Gruene et al., 2015a). While we did not see darting behavior in females during any of the trials, possibly due to rat strain or specific testing conditions that promote or diminish darting, we did see a divergence in rearing between our ER and EC groups that was associated with differences in freezing, suggesting another possible phenotypic predictor of extinction learning in females. This might be supported by a study suggesting that measures of CO_2_ reactivity, including rearing, were predictive of extinction phenotype in male rats (Monfils et al., 2019).

Although EC and ER females showed differences in freezing, rearing, and 50 kHz USVs, the difference in freezing during extinction learning in ER and EC females was not as robust as that seen in males (Sharko et al., 2017; Kellis et al., 2020). This might be attributed in part to different fear behaviors intrinsic to male and female rodents, as suggested by Gruene et al. (2015a) for fear conditioning and extinction, including stress-induced burying, rearing, escape-type behaviors (e.g., darting), or other fear responses as opposed to immobility (Wilson et al., 2004). For example, female rats show significant burying behavior during a witness stress, which is not generally seen in males and is reduced by ovariectomy (Finnell et al., 2018). Previous studies also report that females generally produce less freezing than their male counterparts during various stages of fear paradigms (Maren et al., 1994; Pryce et al., 1999; Gupta et al., 2001; Wiltgen et al., 2001; Chang et al., 2009), and this diminished level of freezing might have blunted the separation between extinction phenotypes in our female population. In contrast, using a fear conditioning protocol that assesses both freezing and flight (escape) responses, females showed greater freezing than males, but no differences in flight responses (Borkar et al., 2020). This is in contrast to the lack of male–female differences in freezing (Gruene et al., 2015b), but higher levels of darting, thought to be a type of escape behavior, seen in females compared to males during fear conditioning and extinction trials (Gruene et al., 2015a). Sex differences in freezing are also seen in paradigms that require discrimination between both fear and safety cues (Clark et al., 2019; Greiner et al., 2019; Borkar et al., 2020) or fear generalization (Jasnow et al., 2017), perhaps suggesting sex differences in the ability to discriminate fear and safety cues or environmental stimuli that might have influenced fear extinction in a novel context in our paradigm. Further, we selected a median split during extinction learning to separate the phenotypes for comparison to our studies in males (Sharko et al., 2017; Kellis et al., 2020), and it clearly led to two phenotypes based on overall freezing, rearing, and 50 kHz USV differences between ER and EC groups. Several prior studies in males or females, however, used extinction recall trials to determine the extinction phenotype (Herry and Mons, 2004; Burgos-Robles et al., 2007; Gruene et al., 2015b). Finally, it is possible that having females at different stages of the estrous cycle might have slightly shifted freezing during extinction trials (Borta et al., 2006; Gruene et al., 2015b; Velasco et al., 2019; Day and Stevenson, 2020) and/or fear generalization (Jasnow et al., 2017), and blunted the distinction in freezing between ER and EC females.

To our knowledge, this is one of the first studies demonstrating that EC females emit more 50 kHz USVs than ER females during fear learning, extinction learning, and generalization trials, especially during the initial exposure to the novel contexts in these trials. Further, the differences in 50 kHz USVs were seen prior to any conditioning during the first 3 min of the fear learning trial. This might suggest differences in exploration or processing of contextual environments between these phenotypes, which could influence the affective perception of the stimuli during fear learning. This phenotypic difference in 50 kHz USV emission is particularly interesting given the lack of freezing differences during fear learning, suggesting separate neural mechanisms may mediate fear-associated freezing and USV emission between EC and ER groups. It could also suggest that ER and EC groups have differences in consolidation of fear memories after the conditioning trial based on potential differences in stress-related responses in a novel environment, thus contributing to the differential levels of fear extinction. This might be supported by the slight (although non-significant) differences between ER and EC groups during the first 5–10 tones of extinction learning supporting a potential difference in cued fear memory recall. There was initially a high number of 50 kHz vocalizations in EC females during the fear learning and extinction trials that decreased over time, particularly with the onset of the tone–shock pairings in fear learning, conditioned tones in extinction learning or recall, or even novel tones in generalization. Similar decreases in 50 kHz emissions have been observed in other studies (Brudzynski and Pniak, 2002; Wöhr et al., 2008; Sangarapillai et al., 2021). Combined with the reduced number of 50 kHz USVs during contextual fear recall, one interpretation is that females emit 50 kHz calls during exposure to a new or safe environment, and these are decreased during fear-inducing stimuli including footshock, conditioned cues, and conditioned contexts. This might suggest that fear behaviors may be expressed as an increase in behaviors associated with fear (freezing, 22 kHz USVs), and/or a decrease in behaviors related to more positive affect (50 kHz calls). The EC rats showed more 50 kHz USVs during exposure to novel environments, plus less reduction during these fear-inducing stimuli, which may be associated with the more effective extinction learning and recall processes that reduce a fearful state in a novel, safe environment. This might be related to the proposed investigative function of 50 kHz USVs and/or associated with phenotypic differences in the more rewarding aspects of novelty-seeking behaviors (Brudzynski, 2021). The 50 kHz USVs have been shown to occur during periods of active sniffing (Sirotin et al., 2014) and to correlate with rearing behavior (Sangarapillai et al., 2021). Thus, it is not surprising that particularly EC rats emit more 50 kHz vocalizations during the exploration periods in all of our trials prior to exposure to conditioned tones or tone–shock pairings, since they also displayed more rears during this period. Nevertheless, the ethograms in Figure 5 did not suggest a close temporal coherence between 50 kHz USVs and rearing (or other) behaviors. Furthermore, ER and EC groups displayed similar rearing but different numbers of 50 kHz USVs before fear conditioning. Another possible explanation for the higher levels of 50 kHz calls seen in EC rats compared to ER rats could be susceptibility to social isolation. Rats socially deprived for 3 weeks display enhanced 50 kHz calls (Hamed et al., 2009), so it is a possibility that our single-housing environment before testing could induce individual variations in 50 kHz USVs in a novel environment and/or perhaps contribute to the phenotypic differences in extinction. Additional studies will be needed to investigate the specific impact of social isolation and/or novelty seeking in the differences in 50 kHz vocalizations in both male and female ER and EC phenotypes, including assessments in different tasks of exploration, anxiety, and/or learning.

In the present study, we found female rats emitted very few 22 kHz alarm-type USVs, agreeing with previously published studies demonstrating fewer 22 kHz USVs in females than males during fear conditioning and fear extinction (Koo et al., 2004; Borta et al., 2006; Litvin et al., 2007; Shumake et al., 2014; Schwarting, 2018a,b; Willadsen et al., 2021). For example, in an auditory conditioning paradigm, it was reported that during fear learning, emission of 22 kHz USVs is significantly influenced by sex, with nearly three times as many males emitting 22 kHz calls during this stage than females (Willadsen et al., 2021). Interestingly, in a comparison of USV emission between females of three different rat strains, Long–Evans rats, the strain used in the present study, produced the most 22 kHz calls (Schwarting, 2018a). Despite this difference in 22 kHz emissions, the authors did not observe inter-strain differences in freezing behavior, concluding potential divergences in physiological or neurobiological mechanisms underlying freezing behavior and USV production; this suggestion is supported by lesion studies of the amygdala (Koo et al., 2004). A similar observation was made by Wöhr et al. (2005), who reported that male rats did not produce 22 kHz USVs in concordance with freezing, positing that USVs must be influenced by factors other than freezing. Our observations of a high number of 50 kHz calls by female rats contrasts greatly with male emissions in this range. USVs by males in the 50 kHz range during fear conditioning paradigms are seldom reported, since they are thought to be associated with more positive stimuli (Koo et al., 2004; Wöhr et al., 2005; Portfors, 2007; Brudzynski, 2021). Contrasting with these rare 50 kHz emissions by males, the female rats in the present study produced a high number of 50 kHz USVs, especially during the initial exposure to the novel environment in different trials. This larger number of 50 kHz USVs by females has previously been observed during the unconditioned context phase of an extinction trial as well as during Pavlovian conditioned avoidance (Sangarapillai et al., 2021; Willadsen et al., 2021). Shumake et al. (2014) reported infrequent 50 kHz calls in both males and females, noting that most occurred at the end of extinction trials (Shumake et al., 2014); this is similar to our results where more 50 kHz USVs were seen during extinction recall, especially in the EC group. Future studies including finer assessments of both 22 and 50 kHz call types and parameters would be helpful in elucidating the functional significance of these differences in USVs between EC and ER females and males (see Brudzynski, 2021). Acoustic features such as call durations, interval lengths, bout lengths, and frequency modulation may reflect subject-specific aspects of the affective state and/or situation (van der Poel et al., 1989; Wöhr et al., 2005; Lenell and Johnson, 2017; Kõiv et al., 2021).

The differences in freezing, rearing and USVs in ER and EC phenotypes may be related to differential regulation by various neuromodulators, neurotransmitters or neural circuits associated with fear extinction, although many of these studies have been done in males rather than females. Differences between extinction phenotypes in rats or mice have been seen in neuronal activation, firing patterns, morphological changes, or synaptic plasticity in the prefrontal cortex (PFC) and basolateral amygdala (BLA), the key nodes in the fear extinction circuit (Herry and Mons, 2004; Burgos-Robles et al., 2007, 2009; Holmes and Singewald, 2013; Gruene et al., 2015b). Evidence suggests that fear responses rely on theta (4–12 Hz) and gamma (30–120 Hz) oscillatory behavior between the BLA and PFC (Likhtik et al., 2014; Stujenske et al., 2014; Bocchio et al., 2017; Dupin et al., 2019), and that 4 Hz oscillations between the PFC and BLA predict fear-related freezing (Karalis et al., 2016). Interestingly, in males there is not only a correlation between respiration and 4 Hz PFC oscillations (Biskamp et al., 2017; Tort et al., 2018), but 22 kHz USV emissions disrupt respiration and correlate with changes in the oscillations between the PFC and BLA (Dupin et al., 2019). Reactivity to a CO_2_ challenge was shown to predict extinction phenotype based on freezing response in males (Monfils et al., 2019), suggesting the relationships between extinction phenotypes, respiratory activity, and oscillatory connections between PFC and BLA in terms of freezing and USVs in males or females remains an intriguing area to be investigated. Extinction resistance in male mice or rats has also been associated with altered regulation of several neurotransmitter or modulator systems, including the glutamatergic system (Burgos-Robles et al., 2007; Holmes and Singewald, 2013), lateral hypothalamic orexin neurons (Sharko et al., 2017; Monfils et al., 2019), and cholinergic signaling (Kellis et al., 2020), however, if these same systems are also involved in extinction phenotypes in females remains to be investigated in future studies. In males, we have shown a correlation between acetylcholinesterase activity in the amygdala, which regulates acetylcholine neurotransmission, and freezing during fear extinction (Kellis et al., 2020). Although the association between acetylcholinesterase activity with USVs during fear extinction was not investigated, the induction of 22 kHz USVs involves activation of the ascending cholinergic afferents from the mesolimbic cholinergic system (Brudzynski and Bihari, 1990; Brudzynski et al., 1991; Brudzynski, 2001, 2007, 2021; Machold, 2013). Emission of 50 kHz USVs is regulated by dopaminergic mechanisms, however, studies suggest that emotional regulation of both 22 and 50 kHz USVs may be due to the interaction between dopaminergic and cholinergic mechanisms (see Silkstone and Brudzynski, 2020; Brudzynski, 2021). The potential role of individual differences in cholinergic and dopaminergic systems in mediating the divergence in freezing or USVs between ER and EC phenotypes, and/or potential sex differences in USVs during fear conditioning remain to be investigated. One of the few studies to directly compare males and females demonstrated that the morphological changes in the PFC–BLA circuit associated with fear extinction were sex-specific, suggesting that there might be distinct neural mechanisms underlying extinction phenotypes in males versus females (Gruene et al., 2015b).

Although estrous cyclicity was assessed in the present study, the study was not designed to determine the influence of estrous cycle stage in female fear extinction. Of note, both ER and EC groups contained animals on different days of the cycle and therefore having differing levels of estrogen and/or progesterone, suggesting that phenotypic differences in fear extinction were not solely based on gonadal hormone fluctuations. Although the difference between ER and EC groups in freezing and USVs seemed independent of estrous cycle phase, since each trial had females at different stages, we cannot rule out the influence of estrous cycle phase on fear behaviors and USVs in this study. For example, estrous cycle may have contributed to the less robust separation between ER and EC groups. Amygdala function in fear learning and extinction is influenced by estrous cycle phase (Blume et al., 2019), and female rats display enhanced extinction learning during proestrus (Gruene et al., 2015b; Blume et al., 2019). Similarly, female rats in a low-estrogen phase that receive estradiol injections before extinction learning show enhanced activity in the central amygdala during extinction and improved extinction memory (Maeng et al., 2017). There is also evidence in the literature that USV emission is influenced by the estrous cycle, with a higher number of 50 kHz USVs seen during estrous compared with diestrous (Thomas and Barfield, 1985; Matochik et al., 1992; Lenell and Johnson, 2021). However, it has been shown that these hormonal associations are not as prominent when rats are isolated, and it is of note that our rats were individually housed thus possibly diminishing the effects of estrous cycle on USVs (Lenell and Johnson, 2021). Nevertheless, although the present study was underpowered to detect estrous cycle associations with freezing, other behaviors, or USV emissions, it is an area worthy of future investigations.

The present study demonstrates that female rats exhibit individual differences in freezing behaviors that, similar to males, dichotomize into EC and ER phenotypes; this difference was accompanied by a divergence in rearing behaviors as well. This study presents the novel finding of individual differences in 50 kHz vocalizations between EC and ER female rats that was observed with exposure to the novel environment even before fear conditioning. Furthermore, the observation that females appear to vocalize less in the 22 kHz range and more in the 50 kHz range compared to males supports the presence of sex differences in the neurobiological mechanisms underlying USV production and freezing during fear learning and extinction, and that freezing behavior and USV emissions may be modulated by distinct brain processes in a sexually divergent manner. These findings suggest the importance of considering and capturing the full behavioral repertoire of fear expression in order to better understand individual susceptibility, especially in females, to disorders such as PTSD.

## Data Availability Statement

The raw data supporting the conclusions of this article will be made available by the authors, without undue reservation.

## Ethics Statement

The animal study was reviewed and approved by University of South Carolina’s Institutional Care and Use Committee.

## Author Contributions

MW conceptualized, visualized, designed and administered the project, acquired the funding, analyzed the data, and wrote and edited the manuscript. ST performed the investigation, analyzed the data, and wrote and edited the manuscript. IS performed the investigation, analyzed the data, and wrote and edited the manuscript. DK performed the investigation and helped edit the manuscript. KK performed the investigation. All authors contributed to the article and approved the submitted version.

## Conflict of Interest

The authors declare that the research was conducted in the absence of any commercial or financial relationships that could be construed as a potential conflict of interest.

## Publisher’s Note

All claims expressed in this article are solely those of the authors and do not necessarily represent those of their affiliated organizations, or those of the publisher, the editors and the reviewers. Any product that may be evaluated in this article, or claim that may be made by its manufacturer, is not guaranteed or endorsed by the publisher.
